# Long-term functional outcome between Yamane technique and retropupillary iris-claw technique in a large study cohort

**DOI:** 10.1097/j.jcrs.0000000000001421

**Published:** 2024-02-08

**Authors:** Pier Luigi Guerin, Gian Marco Guerin, Marco Rocco Pastore, Stefano Gouigoux, Daniele Tognetto

**Affiliations:** From the Eye Clinic, Department of Medical, Surgical Sciences and Health, University of Trieste, Trieste, Italy.

## Abstract

**Purpose:**

To evaluate which secondary intraocular lens (IOL) implantation technique was more successful in achieving the best postoperative results and refractive outcomes between retropupillary iris-claw IOL (ICIOL) and flanged intrascleral IOL (FIIOL) fixation with the Yamane technique.

**Setting:**

Eye Clinic of the University of Trieste, Trieste, Italy.

**Design:**

Retrospective observational study.

**Methods:**

116 eyes of 110 patients who underwent ICIOL or FIIOL were analyzed. Patients with follow-up shorter than 6 months or with incomplete clinical data were excluded. Collected data included demographics, ocular comorbidity, indication of surgery, intraocular pressure, early (≤1 month) and late (>1 month) postoperative complications, corrected distance visual acuity (CDVA), and manifest refraction at the last follow-up visit.

**Results:**

50% (n = 58) of eyes underwent FIIOL and 50% (n = 58) ICIOL implantation for aphakia (n = 44, 38%) and IOL dislocation (n = 72, 62%). No statistically significant differences in demographics, comorbidity, follow-up duration, postoperative complications, and surgical indications were found. The refractive prediction error (RPE) was 0.69 ± 0.94 diopter (D) in the FIIOL group and 0.21 ± 0.75 D in the ICIOL group (*P* = .03), indicating residual hyperopia after both techniques. RPE, mean absolute error, and median absolute error were higher in the FIIOL group (*P* = .003). ICIOL implantation was more successful in obtaining a RPE between −0.50 D and +0.50 D (52% of ICIOL, n = 30, and 31% of FIIOL, n = 18).

**Conclusions:**

Both techniques were effective in increasing preoperative CDVA with no statistical difference between them. Although complication rates did not significantly differ, the FIIOL group exhibited less predictable refractive outcomes. Adjusting the dioptric power of the 3-piece IOL, as performed in ciliary sulcus implantation, to prevent myopic shift, is not recommended.

## Abstract 2

FCIOL fixation is associated with a minor incidence of IOP spikes and CMO, a higher rate of tilt, dislocation, and less predictable refractive outcomes than ICIOL fixation.

In optimal conditions, intraocular lenses (IOLs) are inserted into the capsular bag during cataract surgery immediately after phacoemulsification. However, this approach is not feasible in case of lacking capsular support, necessitating an alternative technique for IOL implantation in these situations.^1^

The main indications for secondary IOL implantation include aphakia, lens or IOL dislocation, incorrect dioptric power of a previously implanted IOL, intolerance to multifocal IOL, IOL opacification and uveitis, glaucoma, and hyphema syndrome.^2^ High myopia, pseudoexfoliation syndrome, and previous vitreoretinal surgery are the most frequently reported risk factors for late in-the-bag IOL dislocation.^1,3^

Before performing a secondary IOL implantation, evaluating the presence of capsular bag support is mandatory. In eyes with adequate capsular support, IOLs can be positioned in the sulcus with or without optic capture. Whereas, in eyes with lacking capsular support, the main options are anterior chamber implantation with angle support, iris-claw, or scleral fixation.^4^ Although anterior chamber implants are less commonly used because of their rate of long-term complications, studies have not conclusively established which technique between iris-claw and scleral fixation yields better functional outcomes, leaving the final decision to the surgeon's experience and skill.^5–8^

This study focuses on 2 widely adopted techniques for secondary IOL implantation in eyes without capsular bag support: retropupillary iris-claw IOL (ICIOL) and flanged intrascleral IOL (FIIOL) fixation as described by Yamane, comparing the final functional outcomes within a relatively large cohort.^9^ The primary outcome was to evaluate the more successful technique in getting a better postoperative corrected distance visual acuity (CDVA) with the lower refractive prediction error (RPE) and mean absolute error (MAE). As a secondary outcome, we analyzed which surgical approach was less burdened by intraoperative and postoperative complications.

## METHODS

In this retrospective observational study, clinical and instrumental data of 116 eyes of 110 patients who underwent secondary IOL implantation with or without concurrent pars plana vitrectomy (PPV) at Eye Clinic of Trieste between January 2019 and May 2022 were collected. The local ethics committee approved this study, and the tenets of the Declaration of Helsinki were followed throughout the study. Informed written consent was obtained from each patient. Indications for surgery were surgical aphakia (38%) and IOL dislocation (62%). None of the enlisted patients had adequate capsular support. Patients were divided into 2 groups according to the surgical procedure they went through FIIOL or retropupillary ICIOL fixation. To limit the learning curve effect, we excluded the first 10 cases of FIIOL implantation performed by our surgeon. The IOLs used for each group were, respectively, the Tecnis ZA9003 IOL (A-constant 119.1) (Johnson & Johnson Vision), and the Artisan Aphakia lens (A-constant 115.7) (Ophtec BV).

### Data Acquisition

All the patients received a complete preoperative examination within 1 week before surgery and subsequent postoperative controls. Exclusion criteria were follow-up shorter than 6 months or incomplete clinical data. The following preoperative baseline data were collected: demographics, indication for surgery, CDVA, intraocular pressure (IOP), and ocular comorbidities (ie. glaucoma, retinal pathologies, previous retinal detachment, and corneal pathologies). We also recorded intraoperative complications and whether PPV was performed. Early postoperative complications were named when occurring within the first month (ie, corneal edema, IOP rising, anterior or vitreous chamber bleeding, IOL dislocation or tilt, cystoid macular edema [CME]) and late complications for those occurring after 1 month from surgery (ie, worsening of glaucoma, CME, retinal detachment, corneal decompensation, and dislocation or tilt). CDVA and manifest refraction were analyzed at the last follow-up visit.

The primary outcome was to evaluate the more successful technique in getting a better postoperative CDVA with the lower RPE and MAE. As secondary outcome, we analyzed which surgical approach was less burdened by intraoperative and postoperative complications. Corneal edema was considered significant if it persisted more than 1 week after the surgery, IOP was measured with Goldmann applanation tonometry, and glaucoma was considered worsened if the increase of the IOP did not resolve within 1 month, requiring the introduction or modification of an already ongoing topical therapy, or when a progression in visual field examination was observed. Tilt was recorded as complication when clinically significant, in particular when clearly detectable at slitlamp examination and associated with optical aberrations; it was also confirmed with ultrasound biomicroscopy. All patients underwent optical coherence tomography (Heidelberg Spectralis, Heidelberg Engineering) scanning to detect the onset of CME during all follow-up visits.

### Refractive Analysis

RPE was obtained by subtracting the postoperative spherical equivalent of each patient from the preoperative refractive target calculated by our biometer (IOL Master 700, Carl Zeiss Meditec AG) using the SRK/T formula for normal and long eyes (≥22.0 mm) and the Haigis formula for short eyes (<22.0 mm). We aimed for either a slightly myopic target (ie, −0.25 diopter [D] spherical equivalent) or a myopic refractive target (ie, −2.50 D spherical equivalent) depending on the axial length and the patients' preferences. MAE is intended as the absolute value of the RPE. Patients with CDVA less than 20/40 at the last follow-up visit were excluded from the refractive analysis and 3-piece refixed IOLs because of the unknown dioptric power and RPE.

For the FIIOL fixation, the dioptric power of the IOL was calculated as in cases of implantation in the ciliary sulcus to prevent the frequently reported myopic refractive results in case of scleral fixation IOLs, reducing the dioptric power according to the indications in the literature.^4,9–13^ To our knowledge, the RPE of modern IOL formulas in the Yamane technique has not still been definitely determined, and only a few studies reporting the refractive outcomes following this fixation technique are available.^9,14^

### Surgical Technique

Surgery was performed in all the cases by the same expert surgeon (D.T.) under retrobulbar anaesthesia. In case of ICIOL fixation, a superior 5.5 mm scleral frown incision was made with paracentesis at 10 and 2 o'clock (Video 1). The dislocated lens was removed through the main incision. Anterior chamber volume during the anterior vitrectomy execution was maintained by injecting the viscoelastic material. The IOL positioning was then executed by holding its plate with a pair of forceps and performing retropupillary enclavation of the iris into the IOL claw using an enclavation needle.

In case of FIIOL fixation through the Yamane technique for eyes with a foldable IOL, it was cut or folded in the anterior chamber and removed through a 2.4 mm corneal incision (Video 2). Subsequently, a 3-piece IOL Tecnis ZA9003 was inserted into the anterior chamber with the trailing haptic prolapsing through the corneal incision. Two symmetrical 2 mm scleral tunnels were created using a 30-gauge bent needle 2 mm posterior to the limbus 180 degrees apart. The haptics were then straightened into the needle lumens and then externalized over the conjunctiva. Low-temperature cautery was used to melt the terminal ends of the haptics creating the flanges which were buried into the scleral tunnels.

All patients received limbal anterior vitrectomy or, if required (ie, in case of IOL luxation in vitreous chamber), 25 or 27-gauge PPV, before proceeding with secondary IOL implantation. In case the dislocated IOL was composed of poly(methyl methacrylate), it was extracted through a scleral frown incision, and the ICIOL technique was favored, yet, if a 3-piece IOL was involved, preference was given to FIIOL fixation.

### Statistical Analysis

For statistical analysis, SPSS for Macintosh software (v. 25.0, SPSS, Inc.) was used, and a probability of less than 5% (*P* < .05) was considered statistically significant. The values reported in this study were expressed as mean ± SD. Data distribution for normality was checked using the Kolmogorov-Smirnov test. Depending on the data distribution, analysis of variance (ANOVA), Mann-Whitney *U* test, or *t* test was used for comparison between groups. The chi-square with the Yates correction test was performed as an independency test between categorical data.

## RESULTS

According to the surgical procedure, patients were divided as follows: 58 eyes in the retropupillary iris-claw fixation group and 58 eyes in the scleral fixation through the Yamane technique group.

Table 1 presents demographic data and preoperative characteristics of the patients. The mean follow-up for FIIOL and ICIOL was 12 ± 4.4 and 14.1 ± 7.7 (*P* = .20) months, respectively. None of these parameters were statistically different in the study groups. In particular, both ICIOL and FIIOL groups achieved a significant improvement in CDVA (*P* < .05): Mean postoperative CDVA was 0.23 ± 0.30 logMAR in the former and 0.21 ± 0.24 logMAR in the latter, with no significant group difference at last follow-up visit (*P* = .76, ANOVA).

**Table 1.** Demographics, preoperative characteristics, and preoperative and postoperative CDVA

Parameter	FIIOL (N = 58)	ICIOL (N = 58)	*P* value
Age (y), mean (SD)	76.2 (9.2)	76.8 (9.8)	.82
Sex (M/F), n (%)	34 (59)/24 (41)	30 (52)/28 (48)	.58
Follow-up (mo), mean (SD)	12 (4.4)	14.1 (7.7)	.20
Preop CDVA (logMAR), mean (SD)	0.61 (0.46)	0.54 (0.46)	.54
Postop CDVA (logMAR), mean (SD)	0.21 (0.24)	0.23 (0.30)	.76

FIIOL = flanged intrascleral IOL; ICIOL = iris-claw IOL

Percentages are calculated as (n/N) × 100

Table 2 presents the indications for surgery according to the technique: Overall, 44 (38%) patients underwent surgery for aphakia and 72 (62%) for IOL dislocation. The surgical technique was decided by the surgeon, and if pars plana vitrectomy was performed, a FIIOL implantation was preferred to avoid further incisions. However, the number of PPV performed in each group did not show statistically significant difference, with 18 and 10 PPV in the FIIOL and ICIOL groups, respectively (*P* = .13, chi-square test with Yates correction). The table also presents patients' comorbidities, with glaucoma being the most frequent and corneal pathologies the least common. The high prevalence of glaucoma in our patients can be attributed to the high prevalence of pseudoexfoliation syndrome in our region, reaching 43% in our study population, and its average age.

**Table 2.** Surgical indication and preoperative patients' comorbidity

Surgical indication	FIIOL	ICIOL	*P* value
Aphakia, n (%)	26 (45)	18 (31)	.18
Subluxation, n (%)	32 (55)	40 (69)	
Comorbidity			
Glaucoma, n (%)	14 (24)	22 (38)	.16
Retinal pathologies, n (%)	14 (24)	17 (29)	.68
RD, n (%)	6 (10)	4 (7)	.74
Corneal pathology, n (%)	0 (0)	3 (5)	.24

FIIOL = flanged intrascleral IOL; ICIOL = iris-claw IOL; RD = retinal detachment

Table 3 presents early postoperative complications. In both groups, the most frequent early complication was IOP elevation, occurring in 12 (21%) and 21 (36%) patients in the FIIOL and ICIOL groups, respectively (*P* = .16). Corneal edema, lasting more than a week, occurred in 2 (4%) and 3 (5%) patients in the FIIOL and ICIOL groups, respectively (*P* = .65). IOL dislocation or tilt requiring repositioning or exchange of the IOL occurred in 7 (12%) patients in the FIIOL group and in 1 (2%) case in the retropupillary iris-claw group (*P* = .07). Hemorrhage, either vitreous or in the anterior chamber, was encountered in 5 (9%) patients in the FIIOL group and in 2 (4%) patients in the ICIOL group (*P* = .61), with each case resolving spontaneously. In the FIIOL group, 2 (4%) patients had intraoperative vitreous hemorrhage during the IOL fixation, requiring PPV; in the ICIOL group, there were no operative complications. No case of hypotony was encountered in either group.

**Table 3.** Early postoperative complications (≤1 month)

Complication	FIIOL	ICIOL	*P* value
Corneal edema (>7 d), n (%)	2 (4)	3 (5)	.65
IOP elevation, n (%)	12 (21)	21 (36)	.16
Hemorrhage (hyphema or vitreous), n (%)	5 (9)	2 (4)	.61
IOL dislocation/tilt, n (%)	7 (12)	1 (2)	.07

FIIOL = flanged intrascleral IOL; ICIOL = iris-claw IOL

In Table 4, late postoperative complications are reported. CME was the most frequent, affecting 8 (14%) and 13 (23%) patients in the FIIOL and ICIOL groups, respectively (*P* = .34). Glaucoma progression was found in 6 (10%) patients in the FIIOL group and in 5 (9%) patients in the ICIOL group (*P* = .75). Three cases of retinal detachment were observed following FIIOL and one after ICIOL fixation (*P* = .61, chi-square test). No cases of pupillary block, corneal decompensation, endophthalmitis, or choroidal effusion were observed.

**Table 4.** Late postoperative complications (>1 month)

Complication	FIIOL	ICIOL	*P* value
Escalation of glaucoma, n (%)	6 (10)	5 (9)	.75
CME, n (%)	8 (14)	13 (23)	.34
RD, n (%)	3 (5)	1 (2)	.61
Corneal decompensation, n (%)	0	0	—
IOL dislocation/tilt, n (%)	0	1 (2)	.32

CME = cystoid macular edema; FIIOL = flanged intrascleral IOL; ICIOL = iris-claw IOL; RD = retinal detachment

Table 5 presents RPE, MAE, and median absolute error (MedAE) at last follow-up visit. A statistically significant CDVA increase was obtained after surgery in both groups without significant differences between them (*P* = .76, ANOVA). The resultant RPE in the FIIOL group was 0.69 ± 0.94 D, while in the ICIOL group, it was 0.21 ± 0.75 D (*P* = .03, ANOVA). This result indicates a tendency toward hyperopia, higher in patients who underwent scleral fixation. Consistently, MAE and MedAE were higher in the Yamane group (*P* = .003, ANOVA). Patients who underwent retropupillary iris-claw implantation were more likely to obtain a refractive outcome within ±0.50 D. The result is similar when considering postoperative refractive outcomes within ±1.00 D.

**Table 5.** Refractive analysis

Parameter	FIIOL	ICIOL	*P* value
RPE (D), mean (SD)	0.69 (0.94)	0.21 (0.75)	.03
MAE (D), mean (SD)	0.98 (0.59)	0.60 (0.49)	.003
MedAE (D)	1.15	0.60	
% of eyes within			
RPE ±0.50 D	31	52	
RPE ±1.00 D	50	83	

FIIOL = flanged intrascleral IOL; ICIOL = iris-claw IOL; MAE = mean absolute error; MedAE = median absolute error; RPE = refractive prediction error

## DISCUSSION

Given the specific advantages and complications associated with each of the 2 analyzed surgical techniques, objectively determining the most suitable approach for patients without sufficient capsular support represents a challenge; therefore, many authors assert that the decision should be based on surgeon's experience and the patient's ocular comorbidities. Newer sutureless techniques have improved scleral fixation by removing the risk of suture erosion or breakage and reducing the surgical time, increasing their diffusion.^15,16^ In this background, we analyzed the functional outcome and the intraoperative and postoperative complications of the FIIOL fixation described by Yamane comparing them with the well-established ICIOL technique in a large study cohort.^9^

The ICIOL boasts the advantages of being a more widespread and relatively simpler surgical technique, requiring less time compared with other implantation methods. However, it comes with drawbacks, including the necessity for a frown scleral incision with sutures, frequent postoperative inflammation such as corneal edema and anterior chamber flare, pigment dispersion, corectopia, and dysphotopsias in case of malposition. In addition, this technique also depends on the iris condition, with atrophy and donesis representing a contraindication if moderate or severe.^7,17^

On the other hand, FIIOL uses a less invasive transconjunctival approach with smaller corneal incisions, does not require the creation of scleral flaps, and positions the IOL in a more physiological location, ensuring less postoperative inflammation and quicker visual recovery. However, it requires more surgical experience and presents a nonnegligible risk of decentration or tilt of the IOL. Compared with other scleral fixation techniques, it offers relatively shorter operating times and does not need a customized IOL.^7,17–19^

In our analysis, the 2 techniques yielded a significant CDVA gain without statistically significant differences between them, although both were associated with different complication rates and postoperative refractive results. As indicated in the results, ICIOL showed a higher rate of IOP increase (31%) than FIIOL (18%) in the first month, although no statistical difference was observed (*P* = .16). Most cases were transient rises occurring in the first postoperative days responsive to medical topical treatment due to a partially retained viscoelastic material, and possibly, mostly in the ICIOL group, to pigment dispersion. In 5 (9%) and 6 (10%) cases in the ICIOL and FIIOL groups, respectively, the IOP rise did not resolve and required an introduction or a modification of an already ongoing topical therapy. No case required glaucoma surgery. Subgroup analysis revealed no difference in early or late-onset IOP increase between glaucomatous and healthy patients (*P* = .56, chi-square test). In the literature, IOP elevation rates range 6% to 28% in ICIOL fixation, while patients who underwent FIIOL are lower ranging 0% to 16%.^3,4,7,9,16,18,20–22^ In our series, higher percentages of IOP spikes may be due to the use of viscoelastic material instead of an anterior chamber maintainer and to the high prevalence of glaucoma and pseudoexfoliation syndrome in the study population.

CME is known to be one of the major causes of a decrease in CDVA after anterior segment surgery.^23^ Reported CME frequency ranges 0% to 25% after ICIOL and 1% to 4% after FIIOL.^3,7,9,11,14,18,21,24,25^ Although the literature suggests favorable CME rates for FIIOL implantation, it is crucial to consider that they are inevitably influenced by the indication for surgery and comorbidities of the cohort; similar considerations apply to corneal edema. In our report, we found 8 (14%) and 13 (23%) CME cases in the FIIOL and ICIOL group, respectively; although they were less in the FIIOL group, we found no statistically significant difference between the 2 groups (*P* = .34). To eliminate the confounding factor of baseline inflammation that may be present in the surgical aphakia group and the role of PPV, a subgroup analysis considering only IOL dislocation as an indication for surgery without PPV was conducted: No cases of CME were found in the FIIOL group (n = 22), while there were 6 in the ICIOL group (n = 38), although the significance of this result may be limited by the restricted sample size (*P* = .13, chi-square test). At the moment of the diagnosis, mean central macular thickness was 530 ± 105 μm and 595 ± 175 μm in the ICIOL and FIIOL groups, respectively, with no statistically significant difference (*P* = .48). Most cases resolved with topical therapy within 6 months. In only 3 cases—1 in the FIIOL group and 2 in the ICIOL group—CME persisted or recurred for more than 6 months. However, each of these patients had a significant retinal comorbidity (epiretinal membrane with tractional edema, high myopia with previous retinal detachment, and nonproliferative diabetic retinopathy, respectively), which could explain the increased severity. Since dexamethasone intravitreal implants are contraindicated when posterior capsule is absent, they were treated with pro re nata intravitreal anti–vascular endothelial growth factor drugs with good results. No case required IOL removal.

A greater rate of early IOL dislocation was observed after FIIOL (12%) rather than in the ICIOL (2%) group (*P* = .07), but we have no reports of late dislocation, suggesting that the intrascleral placement of the haptics may give solid stability to the IOL when performed optimally. Reported rates of dislocation in the literature vary from 0% to 5% in the case of ICIOL and 0% to 6% in the case of FIIOL.^3,7,9,20,24–26^ To enhance IOL stability in the case of intrascleral fixation, some authors have suggested trimming the haptics; this would also reduce the difficulty of introducing them into the needle's lumen and the risk of postoperative extrusion.^27^

The finding of 4 cases of retinal detachments—1 in the ICIOL group and 3 in the FIIOL group—requires careful consideration. In 2 patients, it occurred 8 and 10 months postoperatively, and the other 2 eyes had severe myopia with an axial length greater than 32 mm. Moreover, one of these patients, who underwent PPV + FIIOL fixation for IOL luxation into the vitreous chamber, had previous retinal detachment in the fellow eye. This last case suggests that in patients with risk factors, the causes of retinal detachment may be different, and PPV may not be sufficient to prevent it. With these premises, it is difficult to establish a causal relationship; however, retinal detachment remains a rarely reported complication after these surgeries, performing a meticulous anterior vitrectomy may be useful to avoid vitreous entanglement on the IOL and may reduce this risk.^3,4,8,9,11^

Regarding the postoperative refractive status of the patients, both techniques have shown a tendency toward a hyperopic shift, more pronounced in cases of FIIOL. This is mainly due to the reduction in the dioptric power of the IOL calculated as in cases of implantation in the ciliary sulcus to prevent the frequently reported myopic refractive results in the cases of scleral IOL fixation.^4,9–11^ The high variability of the refractive results can further be explained by the high number of variables that distinguish this technique and affect the effective position of the lens such as the distance of the sclerotomies from the limbus, their inclination, the type of IOL, the white-to-white, the manipulation of the haptics, and the not exactly predictable shortening caused by cauterization.^28,29^ Recently, some improvements have been proposed to obtain more standardized sclerotomies and lens positioning.^30^

MAE resulted lower in the ICIOL group, confirming the greater reliability of this technique concerning the final refractive outcome. Indeed, 52% (n = 30) of patients who underwent ICIOL achieved a RPE between −0.50 D and +0.50 D vs 31% (n = 18) in the FIIOL group. Some authors reported a myopic shift in patients operated with the Yamane technique, suggesting that the IOL power should be lowered by 0.5 diopters from that for in-the-bag fixation, while others obtained hyperopic results like us, suggesting to aim for a slightly myopic target.^14,28^ Considering the numerous variables involved when performing this surgery, we suggest that each surgeon should analyze his results to optimize the personal A constant.

To our knowledge, in the literature, there are only few studies directly comparing the iris-claw and the flanged intrascleral fixation as described by Yamane with a large sample size. In addition, to reduce bias related to the execution of the surgical technique in our series, all the surgeries were performed by the same surgeon. Still, the retrospective nature, the lack of anterior segment OCT report, and endothelial cell loss analysis may represent some limitations.

ICIOL fixation is widely used as a technique to correct aphakia thanks to its efficacy and relative simplicity, while FIIOL is less invasive and allows to place the IOL in a more physiological position. Both techniques were effective in improving preoperative CDVA of the patients, with no statistically significant difference between them. Complication rates did not significant differ, although the FIIOL group exhibited less predictable refractive outcomes. Adjusting the dioptric power of the 3-piece IOL, as performed in ciliary sulcus implantation, to prevent myopic shift is not recommended. Mastering this technique takes longer, but the incidence of these complications can be diminished with experience and can be particularly advantageous in younger patients or in cases where an ICIOL may be contraindicated.

**WHAT WAS KNOWN**

- Iris-claw and flanged intrascleral fixation are 2 diffuse surgical options to treat aphakia in eyes without capsular support.
- There is not strong evidence supporting the superiority of one of the abovementioned techniques over the other in surgical success, surgical complications, and refractive outcomes.

**WHAT THIS PAPER ADDS**

- A direct comparison between iris-claw and flanged intrascleral IOL fixation techniques is performed by the same surgeon in a relatively large cohort.
- Iris-claw fixation is associated with a lower postoperative refractive error.
